# Perioperative Management of Patient with Esophageal Carcinoma and Crigler-Najjar Syndrome Type 2: A Case Report

**DOI:** 10.3389/fsurg.2022.889753

**Published:** 2022-04-27

**Authors:** Dehua Ma, Fang Chen, Xiaoyun Chen, Yu Chen

**Affiliations:** Department of Chest Surgery, Taizhou Hospital of Zhejiang Province Affiliated to Wenzhou Medical University, Linhai, China

**Keywords:** Esophageal carcinoma, Crigler-Najjar syndrome type 2, case report, esophageal squamous cell carcinoma, surgery, perioperative management

## Abstract

**Background:**

Crigler-Najjar syndrome type 2 (CNS-II) is a rare genetic disease that is associated with a lack of uridine diphosphate-glucuronosyltransferase. Esophageal carcinoma is the sixth most common cause of cancer-related death worldwide, for which surgery is the most effective treatment. Reports on patients with both conditions requiring surgery are limited and The impact of hyperbilirubinemia caused by CNS-II on the perioperative period is unknown. Previous studies have found that patients with Crigler-Najjar syndrome have an increased risk of gallstones and related complications, which also poses corresponding challenges to the treatment. Herein, we present a patient with CNS-II who underwent successful thoracoscopic surgery for esophageal carcinoma.

**Case summary:**

A 65-year-old male presented to our hospital with a choking sensation after eating. A physical examination showed yellowing of the sclera and skin. The patient manifested persistent jaundice since birth and had visited many hospitals, but the cause remained undiagnosed. We performed genetic testing, which confirmed CNS-II. Gastroscopy indicated esophageal carcinoma. A multidisciplinary team discussion was carried out to determine the appropriate treatment and perioperative management for this patient. The results show that surgical resection was the most appropriate approach. Finally, the patient underwent thoracoscopic surgery for esophageal carcinoma without complications.

**Conclusion:**

Esophageal carcinoma in patients with Crigler-Najjar syndrome is a rare case, and perioperative management is key in the treatment process. It is necessary to pay close attention to the changes of the disease to prevent complications.

## Introduction

Crigler-Najjar syndrome type 2 (CNS-II), also called Arias syndrome, is caused by mutations in *UGT1A1* and is associated with a deficiency in uridine diphosphate-glucoronosyltransferase (UDP-GT) (1, 2). The disorder is transmitted by autosomal recessive inheritance and is characterized by hyperbilirubinemia (3). Existing studies have shown that patients with Crigler-Najjar syndrome (CN) are at increased risk for gallstones and related complications (4–8), and the early diagnosis of gallstones and cholangitis is challenged due to permanent jaundice. Esophageal carcinoma is an aggressive disease for which surgery is the most effective treatment. However, esophageal carcinoma with simultaneous CNS-II is exceedingly rare, with only a few cases reported thus far. The impact of hyperbilirubinemia on surgical treatment of esophageal cancer is unclear, and its associated complications are unknown. In this paper, we report a patient with CNS-II who underwent successful thoracoscopic surgery for esophageal carcinoma.

## Case Presentation

### Chief Complaints

A 65-year-old male patient experienced a choking sensation during eating for 2 months.

### History of Present Illness

A 65-year-old male patient was admitted to our hospital because of a choking sensation during eating for 2 months, which was accompanied by retrosternal pain and had no obvious cause.

### History of Past Illness

The patient had a 3-year history of hypertension and took telmisartan for blood pressure control. The patient had manifested yellow discoloration of the skin and sclera since childhood, for which he had visited many hospitals, but his condition did not improve with medication.

### Personal and Family History

**Figure 1:**
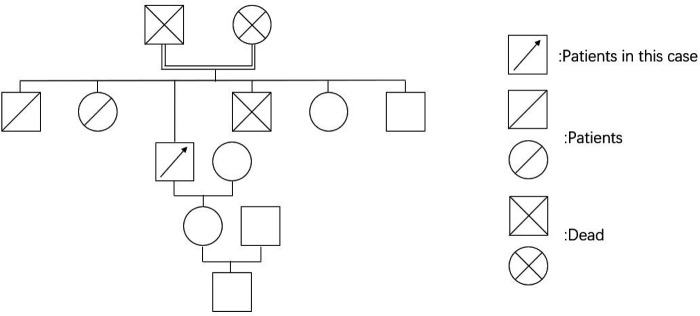
The pedigree map of the patient’s family.

The patient’s parents were of a consanguineous marriage and had raised three sons and three daughters. Two sons and one daughter also had yellow discoloration while the others showed no relevant symptoms. The patient’s daughter and grandson have no relevant symptoms. Its pedigree is shown in **Figure 1**.

### Physical Examination

The patient was generally in a good condition with no supraclavicular lymph node enlargement. The breath sounds of the lungs were rough without rales or Rhonchi. Heart rate was 102 beats/min, and heart rhythm was regular, showing no pathologic murmur. The abdomen was flat and soft, without tenderness or rebound tenderness. Yellow discoloration of the skin and sclera was observed.

### Laboratory Examinations

**Figure 2:**
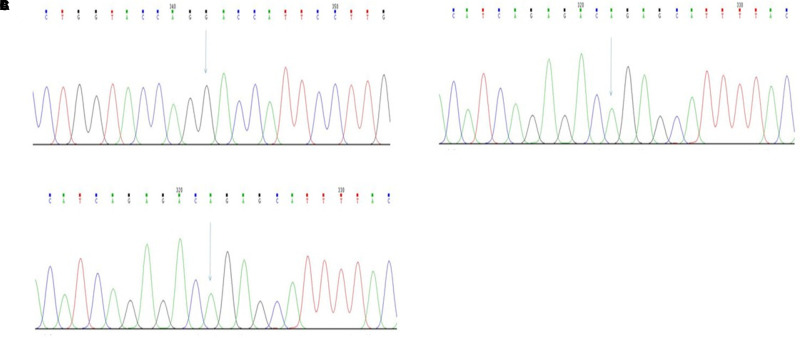
(**A**) ***UGT1A8***_ex5c.1447T > G(p.Tyr483Asp)NCBI reference sequence: CTGGTACCAG TACCATTCCTTG; (**B**) ***UGT1A1***_ex1c.211G > A(p.Gly71Arg) NCBI reference sequence: CATCAGAGAC GGAGCATTTTAC; (**C**) ***UGT1A4***_ex1c.395T > C(p.Leu132Pro) NCBI reference sequence: GTGGAGCTAC TGCATAATGAGG.

White blood cell count was 7.0 × 10^9^/L, neutrophil percentage was 63.1%, hemoglobin concentration was 125 g/L, alanine transaminase was 9 IU/L, aspartate aminotransferase was 18 IU/L, total bilirubin was 187 µmol/L, direct bilirubin was 19.9 µmol/L, and indirect bilirubin was 167.8 µmol/L. Electrocardiography revealed sinus rhythm. we also take a DNA analysis,Genetic testing showed (**Figure 2**):

(1) UGT1A8_ex5 c.1447T > G(p.Tyr483Asp)
(2) UGT1A1_ex1 c.211G > A(p.Gly71Arg)
(3) UGT1A4_ex1 c.395T > C(p.Leu132Pro)

It was suggested that the patient has CNS-II. Other laboratory indicators were all within normal limits.

### Imaging Examinations

**Figure 3:**
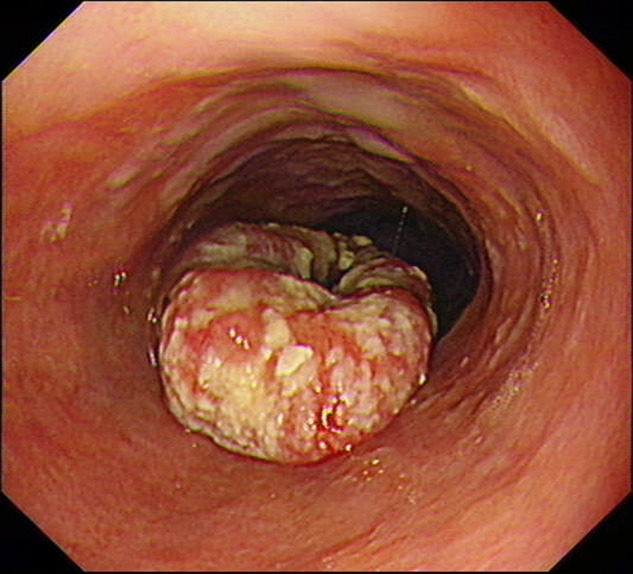
Gastric endoscopy showed the lower part of the esophagus.

Esophagography showed mucosal damage in the mid-to-lower segment of the esophagus, with a filling defect shadow and irregular small niche with a lesion measuring approximately 8.1 cm in length. Gastric endoscopy showed a cauliflower-like ulcero-proliferative growth in the lower part of the esophagus, which was considered to be esophageal squamous cell carcinoma according to the symptoms of the ulcerating surface covered with white moss, and the depressed central part (**Figure 3**).

## Final Diagnosis

The final diagnosis was esophageal carcinoma and Crigler-Najjar syndrome type 2.

## Treatment

**Figure 4:**
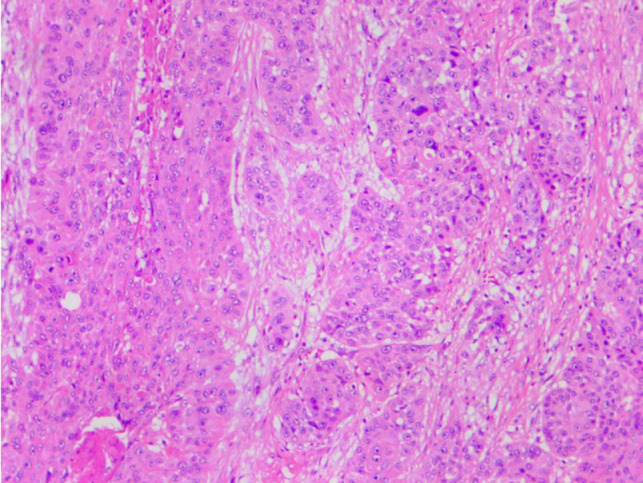
Microscopic findings show squamous carcinoma, (hematoxylin and eosin staining, magnification × 100).

The patient underwent thoracoscopic surgery. Tissue was obtained and sent for pathological examination. The postoperative pathological diagnosis was esophageal squamous cell carcinoma (tumor volume, 7.4 × 5.1 × 1.6 cm). Moderate differentiation was observed with infiltration to the adventitia, and there was no lymph node metastasis (**Figure 4**).

## Outcome and Follow Up

Three months after surgery, the patient revisited our hospital for review. He had recovered well with no related complications, suggesting that the efficacy of surgical treatment was satisfied for this patient with esophageal cancer and CNS-II.

## Discussion

Crigler-Najjar syndrome (CN), which was first described in 1952, is caused by lack of UDP-GT. It can be divided into two categories: type 1 and type 2. Patients with severe jaundice and no response to phenobarbital are classified as Crigler-Najjar syndrome type 1 which type 2 (9).

Crigler-Najjar syndrome type 2 is the less severe form of the disease, with the deficiency in UDP-GT being <30%. UDP-GT is a membrane protein binding on the endoplasmic reticulum, which catalyzes the transfer of the D-Glucuronic acid of UDP – D-Glucuronic acid to other molecules. So it plays play a key part in the Metabolism of bilirubin and promotes the water solubility of acceptors, which promotes the bilirubin excreted from the body by the bile and urine. CNS-II is mostly observed in infants or later in childhood, with bilirubin levels in the region of 102.6–342 mmol/L (10). These patients seldom develop central nervous system involvement complicated by kernicterus. Generally, there are no other notable biochemical parameters, except serum bilirubin. These patients usually survive into adulthood. If the condition becomes serious, it can be treated with phenobarbital to control bilirubin at an appropriate level (11).

Through genetic testing, the patient found three mutated genes, namely UGT1A8, UGT1A1 and UGT1A4. UGT1A8 is a homozygous missense mutation, which has been reported to be detected in Gilbert syndrome and hyperbilirubinemia (12, 13). In vitro functional experiments showed that the mutant protein produced by the mutation had very low scavenging activity against total bilirubin glucuronic acid (13). ClinVar database recorded the mutation as a pathogenic or suspected pathogenic mutation (14). The genetic testing company used SIFT and Polyphen-2 to predict the function of the protein, and the results were both harmful, so the mutation was considered as pathogenic. UGT1A1 is a common variant in Gilbert syndrome and Crigler-Najjar syndrome in the East Asian population (15). In vitro studies have shown that the mutation leads to decreased enzyme activity and is associated with elevated serum bilirubin levels in infants (16). UGT1A1 gene related to CNS-II, Gilbert syndrome (GS), Crigler-Najjar syndrome type 1 (CNS-1) were inherited by autosomal recessive inheritance. As for UGT1A4, ClinVar database has not included this locus, and it is an unknown mutation of clinical significance according to software analysis.

The incidence of CN is approximately at 0.6 patients per million. It is extremely rare for patients with esophageal carcinoma and CNS-II. To the best of our knowledge, this is the first case ever reported. the influence of CN on the occurrence of esophageal cancer is not very well understood, the association CN and early esophageal cancer seen in this case may be incidental. So, there is no experience in this case.

Pre-operative multidisciplinary discussion was held. We all agree that it is important for patients not to suffer starvation for a long period time, which may increase the level of bilirubin. Postoperative vomiting should be avoided by maintaining basal glucose infusion. Ondansetron appears to be efficacious in preventing severe vomiting, but furosemide, salicylates, ampicillin, sulfonamide, and ceftriaxone should be avoided (17). The efficacy of jaundice drugs in the patient was poor, but the jaundice could be reduced. Hepatic encephalopathy may also occur as a postsurgical complication. If necessary, plasma exchange should be performed. Should considerable pruritus occur, phenobarbitals can be used. postoperative parenteral nutrition was administered cautiously to avoid exacerbating jaundice. Should jaundice worsen, bilirubin adsorption is recommended. chemotherapy had a great impact on this patient’s liver function, and radiotherapy alone was not adequately effective in treating esophageal cancer, so surgery was the best choice. In terms of surgical options, we chose minimally invasive surgery, which has a similar overall survival rate, lower complications and better tolerance compared with open surgery (18).

**Figure 5:**
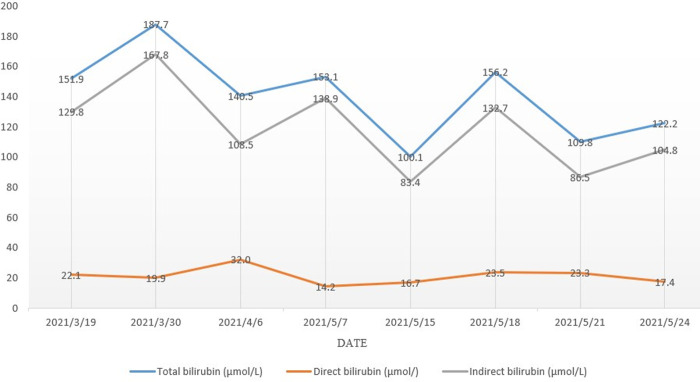
Changes in the bilirubin during treatment.

Surgery went smoothly, and due to concern for the potential impact on liver function, enteral nutrition rather than parenteral nutrition was adopted. During postoperative hospitalization, certain indicators, including liver function, total bilirubin, direct bilirubin, and indirect bilirubin, were similar to those before surgery (**Figure 5**). The patient was able to drink and eat one week after surgery, and he reported no obvious discomfort. He was discharged two weeks after surgery, without much change in various indicators compared with before surgery. Three months later, the patient revisited our hospital for review and had recovered well, with no related complications. This suggests that the efficacy of surgery was satisfied in this patient with esophageal carcinoma and CNS-II.

CNS-II is a relatively benign disease. The management of the disease involves a variety of methods, including lifelong diet adjustment counseling, ensuring adequate hydration, avoiding triggers such as stress, and lifelong phenobarbital treatment. Genetic counseling, especially about blood relationship, is an important part of management and regular follow-up. For the part of esophageal cancer, the patient has successfully received surgical treatment, and according to the postoperative pathological results, the operation has reached R0 resection, and no lymph node metastasis has been found. It is pT1BN0M0 stage IB, does not need postoperative radiotherapy and chemotherapy, and needs regular follow-up and nutritional support treatment.

## Conclusion

Patients with Crigler-Najjar syndrome are at increased risk of various complications due to the presence of persistent hyperbilirubinemia, and the risk of surgical resection may be high, but for diseases that require surgical treatment, surgical treatment can still be performed under close perioperative monitoring and appropriate management.

## Data Availability

The original contributions presented in the study are included in the article/supplementary material, further inquiries can be directed to the corresponding author/s.
